# A Comprehensive Review of Food Safety Culture in the Food Industry: Leadership, Organizational Commitment, and Multicultural Dynamics

**DOI:** 10.3390/foods13244078

**Published:** 2024-12-17

**Authors:** Ashwini Sadashiv Pai, Swarna Jaiswal, Amit K. Jaiswal

**Affiliations:** 1 Centre for Sustainable Packaging and Bioproducts (CSPB), School of Food Science and Environmental Health, Technological University Dublin-City Campus, Central Quad, Grangegorman, D07 ADY7 Dublin, Ireland; d23124799@mytudublin.ie (A.S.P.); swarna.jaiswal@tudublin.ie (S.J.); 2 Sustainability and Health Research Hub, Technological University Dublin-City Campus, Grangerorman, D07 H6K8 Dublin, Ireland

**Keywords:** food safety management systems, organisational leadership, cultural diversity, regulatory compliance, employee engagement, food safety compliance

## Abstract

Food safety culture (FSC) has transitioned from a narrow compliance-based concept to a comprehensive organisational value that is essential for ensuring food safety. This review explores the pivotal roles of leadership, organisational commitment, and cultural diversity in shaping an effective FSC. It highlights how leadership style, particularly transformational leadership, can enhance employee engagement and foster a proactive safety culture. Additionally, the impact of national and organisational cultures on FSC is analysed, emphasising the challenges posed by a multicultural workforce in standardising food safety practices. This article also provides a comparative analysis of FSC across various sectors, such as meat and dairy processing, identifying sector-specific challenges and best practices. In particular, high-risk sectors tend to exhibit a stronger FSC due to regulatory pressure, while other sectors struggle with issues like communication and employee ownership. The importance of integrating behavioural training with cultural considerations is underscored as a key strategy for sustaining a positive FSC. For maintaining a strong FSC, tailored approaches, which account for cultural and operational differences, are necessary for improving food safety outcomes. This comprehensive analysis provides valuable insights for industry professionals and policymakers, offering a foundation for future research and the development of more effective food safety management practices.

## 1. Introduction

Food safety in the manufacturing sector has changed significantly over time, moving from simple hygiene procedures to the complex, methodical methods that are in use today. The concept of FSC came into being as a result of major food safety incidents that raised public awareness and demonstrated the importance of taking preventative measures to ensure food safety. The limitations of the current food safety measures were brought to light by high-profile cases such as the European *bovine spongiform encephalopathy* (commonly referred to as “mad cow disease”) scare, and the *E. coli* outbreaks connected to brand names like Jack in the Box and Odwalla. These incidents sparked a change in thinking that placed more emphasis on building a strong food safety culture within companies as well as adhering to food safety regulations [1].

A major step towards standardising food safety standards was taken in 2000 with the introduction of the Global Food Safety Initiative (GFSI). Although the GFSI was primarily concerned with standardising food safety standards, it also referenced the need to foster a strong FSC. When FSC was first established, it was primarily thought of as a compliance issue, with safety regulations and standards being followed. But, this perspective has changed over time to acknowledge that FSC is about establishing food safety as an organisational core value and shared responsibility, which far exceeds simple compliance [2].

Since the early 2000s, when the idea of FSC first surfaced, it has undergone substantial change. At first, FSC was thought to be nothing more than following guidelines and standards for food safety [1]. The adoption of FSC as a component of quality management systems in food processing industries as well as the integration of behavioural sciences to understand employee motivations and attitudes towards food safety were significant turning points in the evolution of FSC. Developing a strong FSC is particularly crucial to empower employees to act decisively during critical situations, addressing hazards beyond routine procedures. Food safety culture is a long-term construct existing at the organisational level, relating to the deeply rooted beliefs, behaviours, and assumptions that are learned and shared by all employees, which impact the food safety performance of the organisation. This underscores the importance of FSC in shaping both employee behaviour and overall food safety outcomes [3]. Another important area of development has been the role that leadership plays in creating and maintaining FSC. In addition to establishing rules and guidelines, leaders must also set an example for desired behaviour and cultivate an atmosphere in which everyone takes responsibility for food safety [4]. This highlights that FSC is deeply embedded in an organisation’s culture and reflects how “things are done” internally, with a focus on trust, collaboration, and collective accountability [5]. The integration of FSC into regulatory frameworks, such as the GFSI, ensures continuous alignment of food safety practices with scientific advancements, ultimately contributing to global food security [2]. Food safety is now viewed as a core value rather than merely a legal requirement, as part of a wider organisational ethos that has developed over time [4].

This viewpoint is further supported by the New Era of Smarter Food Safety Blueprint, introduced by the U.S. FDA (Food and Drug Administration), which outlines a comprehensive, technology-driven approach to modernise food safety practices and systems. The integration of modernised traceability, proactive prevention approaches, and technological advancements is highlighted, with a strong emphasis on creating a widespread culture of food safety. This blueprint reflects an understanding that a cultural commitment to safety at every level of an organisation, rather than just adhering to standards, is necessary to ensure food safety in the modern era [6]. The ability to take decisive action during critical situations and unforeseen hazards, where routine procedures may fail, further underscores the importance of a strong FSC [5].

As of March 2021, Irish food businesses have been mandated by law to establish, maintain, and demonstrate an appropriate FSC, aligning with the global evolution of food safety standards. This legal requirement stems from the amendment of EU food hygiene regulations, specifically Regulation (EU) 2021/382, which underscores the increasing global recognition of FSC as a critical component for ensuring consumer safety and enhancing public health outcomes [7].

Ireland’s specific regulatory environment and the structural diversity of its food industry present unique challenges and opportunities in implementing FSC. The Irish food sector encompasses diverse industries such as dairy, meat processing, and fresh produce, each influenced by varying organisational cultures and an increasingly multicultural workforce [8]. This diversity necessitates tailored approaches to fostering FSC that consider the distinct operational contexts and cultural dynamics within each sector. This diversity necessitates tailored FSC approaches that account for national cultural characteristics and the operational dynamics within specific sectors. Research also highlights the importance of integrating FSC into an organisation’s social capital by building trust, shared norms, and cooperative networks to support food safety performance [5].

Ireland’s FSC regulations align with international standards such as the Codex Alimentarius and certifications like FSSC 22,000 and IFS, which emphasise management commitment, employee training, efficient communication, and continuous improvement [9]. To effectively operationalise these norms, nevertheless, the Irish context necessitates more attention to incorporating national cultural characteristics and resolving issues unique to the sector. Educational approaches have also evolved, emphasising broader dimensions beyond formal training. One educational framework, for instance, incorporates employee perceptions, relationships, and social interactions to enhance food safety behaviour effectively [10].

While national culture is commonly accepted as having a key role in determining organisational culture and, by extension, FSC, the impact of national culture on FSC has frequently been neglected in previous studies [11]. This article examines the key elements and challenges of establishing a strong food safety culture (FSC) within the food industry, emphasising the role of leadership, organisational commitment, and the impact of cultural dynamics.

## 2. Conceptual FSC Framework

The components of the FSC within the food industry are multifaceted and work together harmoniously, similar to jigsaw puzzle pieces.

Culture surrounding food safety is greatly influenced by the **conduct of management.** Leadership, comprehension, and dedication are necessary for putting food safety protocols into practice. Top management’s influence over work practices and resource allocation has a significant impact on organisational culture [12]. Therefore, management’s proactive involvement in promoting a safety-centric ethos is necessary for creating an effective FSC.

The foundation for the culture of food safety is provided by the organisation’s **vision and mission**. The significance of food safety must be subtly conveyed by these components in clear and consistent corporate communications. Organising efforts towards safety initiatives requires a strategic approach to food safety, which is demonstrated in policy statements and resource allocation [13] (pp. 11–13).

**People** are the foundation of the culture of food safety. Roles and responsibilities must be clearly defined because employee behaviour affects the safety of food products. In order to empower staff members and foster a culture of ongoing improvement in food safety procedures, training, governance, and effective communication are essential [14] (pp. 15–19).

Aligning resources, technology, and processes through **consistency** in food safety practices ensures that food safety programs are implemented effectively. Accountability, compliance, performance evaluation, and clear safety communications are all part of this and are necessary to keep a constant safety culture [13] (pp. 21–23).

Being **adaptable** in the face of shifting circumstances and hazards is essential. A robust culture pertaining to food safety demonstrates a readiness to adjust to any changes, including crises such as product recalls. Furthermore, identifying and mitigating risks to food safety require a thorough **understanding of hazards** at all organisational levels, which can be strengthened through training and metrics [13] (pp. 26–30).

A strong FSC positively impacts food safety by enhancing regulatory adherence and reducing food safety incidents. Organisations with mature FSC have reported measurable improvements in contamination prevention, hygiene standards, and employee engagement [3]. These outcomes stem from the embedding of FSC principles into operational frameworks, which not only ensure compliance with international standards but also foster a culture of continuous improvement. The transformational aspect of FSC is emphasised, where fostering proactive risk management attitudes amongst employees helps mitigate foodborne outbreaks and ensures the sustainability of food safety systems [5].

**Figure 1:**
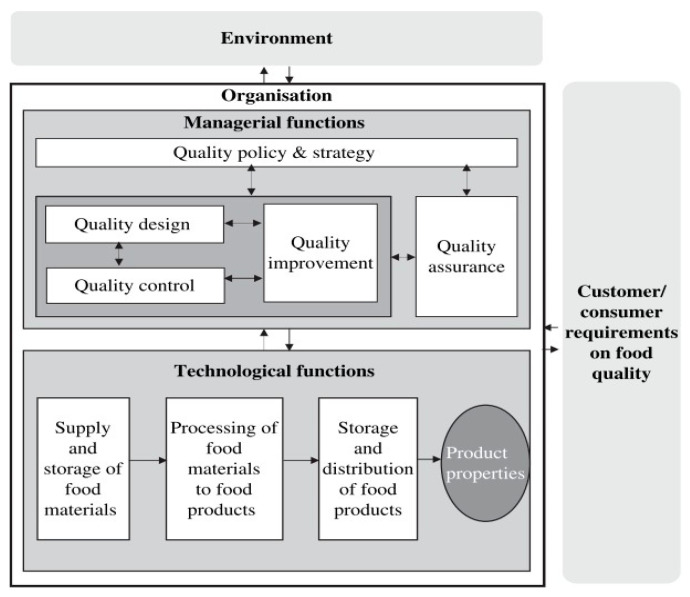
Model of FQM functions [14].

Several concepts and frameworks must be integrated in order to fully comprehend FSC. A food quality management (FQM) model was developed (refer Figure 1), which highlights the interplay between managerial and technological roles in the production of food that satisfies consumer criteria [14]. To guarantee that food production complies with safety regulations, managerial decision-making procedures collaborate with technological activities including heating, storing, and evaluating items.

Based on this, a paradigm that separates FSC into three domains, individual human dimensions, human organisational dimensions, and food safety management system (FSMS), was developed [15]. The organisational block draws attention to elements like risk awareness and leadership, whereas the FSMS concentrates on assurance and control operations. Motivation and compliance are two examples of factors in the individual block that highlight how organisational and human characteristics affect FSC.

**Figure 2:**
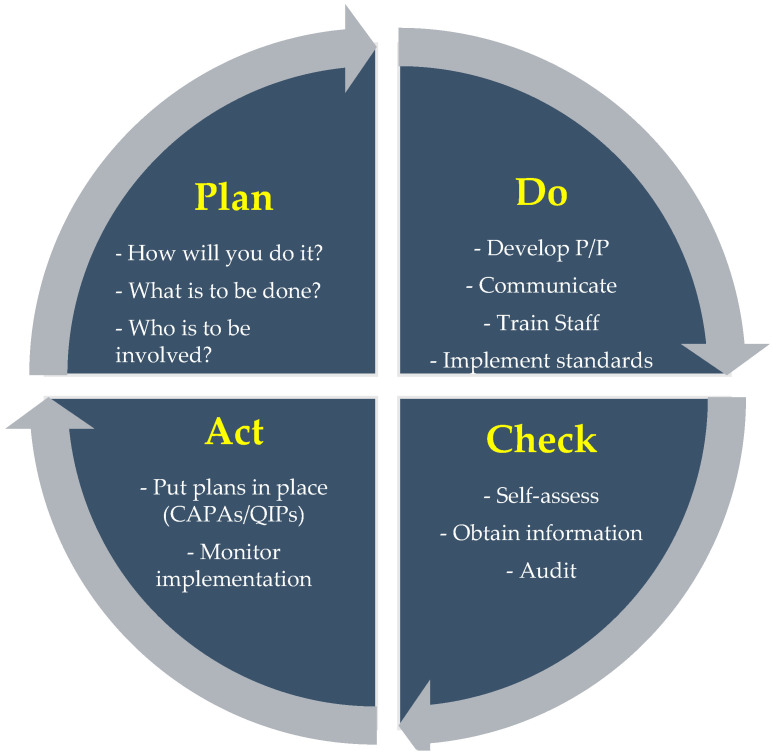
Framework for sustaining and improving FSC [18].

This understanding is further strengthened by the ISO 31000 Risk Management Framework, which provides an organised method for integrating and maintaining a strong FSC [16]. This framework is based on the plan–do–check–act (PDCA) cycle (Figure 2), where management is committed to creating a risk management plan that is specific to the needs of the organisation. Adopting strategies that support FSC objectives is known as implementation (do), and ongoing monitoring (check) makes sure these strategies continue to be successful and adhere to changing standards. Finally, the act phase focuses on making necessary adjustments for continued improvement. This strategy integrates risk management into the everyday activities, reinforcing a dynamic and responsive FSC [16].

The combination of these models underscores the complexity of FSC, demonstrating that a thorough understanding requires consideration of technological processes, organisational dynamics, and risk management principles. The ISO framework, in particular, highlights the importance of ongoing risk assessment and adaptation to maintain high food safety standards.

Additionally, the importance of a strong FSC within organisations cannot be overstated. The cornerstones of food safety and regulatory compliance are the FSMS, which is made up of protocols, procedures, and policies. But, the FSMS covers more than just functional aspects; it also takes into account how people interact with protocols, processes, and products [17]. This emphasises how important it is to map and enhance human behaviour to sustain efficient risk management for food safety.

Unexpected food safety hazards, processes, products, or human error can all lead to FSMS failures. This suggests that addressing the shortcomings of FSMSs requires a robust FSC. The significance of knowledge and comprehension in food safety is emphasised by the application of a Hazard analysis critical control point (HACCP) approach. But, as problems like poor food handler practices, carelessness, noncompliance, and lack of accountability show, knowledge is not enough to guarantee food safety [19].

Additionally, studies highlight the drawbacks of depending only on third-party audits and inspections to guarantee food safety [20]. These approaches, which are frequently seen as momentary glimpses, might not accurately represent an organisation’s daily activities and cannot ensure performance in the future. This has prompted scholars to call for a more integrated approach that incorporates behavioural, social, and psychological change concepts into FSMSs [20].

A paradigm shift in food safety management has occurred with the growing recognition of FSC as a critical component of an organisation’s food safety performance. Human factors are increasingly recognised as significant determinants, which is directing attention towards building a robust FSC [17]. This entails creating an atmosphere where norms, values, and attitudes affect how motivated staff members are to use their education and experience in the workplace [21].

There is a substantial and complex correlation between an effective FSC and organisational performance in the food industry, as presented in Table 1. Studies have provided a crucial illustration of this relationship, demonstrating that companies with a strong FSC typically have higher levels of overall operational efficiency [22] (pp. 11–27). This is partially due to the fact that strong FSCs are frequently associated with lower absenteeism rates and higher employee engagement levels, both of which promote more seamless and effective operations.

Furthermore, fewer food safety incidents are associated with effective FSCs, which has a direct effect on an organisation’s financial performance. For example, a study found that businesses with a strong FSC had fewer recalls and legal issues pertaining to food safety, which helped them save money and improve their reputation [23]. This is particularly important in a sector where a single food safety incident can have a significant financial impact as well as cause long-term brand damage and where maintaining customer trust is crucial.

FSC plays a crucial role in strengthening food safety and security by improving regulatory frameworks and reducing foodborne incidents. Empirical studies have demonstrated the effectiveness of interventions such as implementing food safety key performance indicators (KPIs) and structured group discussions in fostering FSC maturity [3]. These measures help align employee actions with organisational goals, fostering accountability and shared responsibility for food safety outcomes. Organisations with a developed FSC are more likely to abide by industry rules and standards [23]. In addition to helping these organisations stay out of trouble with the law, compliance helps them project a positive image to the marketplace, which frequently results in improved collaborations and business prospects [24].

**Table 1:** Outcomes of strong FSC on organisational performance.

Outcome	Studies	Reference
Compliance with regulations	Strong FSC increases regulatory compliance, reducing penalties and boosting market reputation. In China, post-2008 reforms improved dairy industry compliance. In Greece, co-regulation and strict EU adherence enhanced proactive FSC and food safety performance.	[11]
Financial performance	Investment in a mature FSC enhances safety and reduces contamination risks and overall costs by lowering failures and quality-related expenses.	[25]
Fewer food safety incidents	Significant food safety incidents have revealed the importance of a robust FSC in reducing risks. Effective scientific practices, strong management, communication, and total staff commitment are essential for minimising incidents.	[24]
Employee engagement	To enhance employee engagement, organisations should create supportive environments through programs aimed at existing staff, reducing absenteeism and turnover, thereby boosting satisfaction, retention, and safety reporting.	[26,27]

## 3. Cross-Cultural Considerations

The coexistence of various national cultures within organisations as a result of globalisation has complicated organisational culture and may have an impact on the efficacy of FSMS. The influence of national culture has received little attention, despite the fact that safety outcomes for migrant workers are known to be impacted by communication quality and safety knowledge. Neuroscience studies have revealed startling disparities in basic cognitive processes amongst cultural groups, which may be responsible for increased errors and injuries as well as other problems with safety performance. In particular, disparities between Asian and Western cultures have been noted in risk assessment and risk-taking behaviour, highlighting the significance of taking national cultural influences into account when guaranteeing food safety in organisations [28].

FSC varies significantly across countries and organisations, being shaped by socioeconomic conditions, organisational practices, and cultural dynamics. A study on Lebanese food companies revealed that larger firms and exporters achieve higher FSC scores due to better resources and adherence to global standards. In contrast, frontline employees in smaller organisations often report weaker FSC perceptions, which can be attributed to limited training opportunities and leadership support. External pressures such as economic crises and the COVID-19 pandemic further influence FSC by testing organisational resilience [29].

In low- and middle-income countries (LMICs), informal markets dominate the food supply chain, providing affordable food options but often lacking structured safety protocols. Despite resource constraints, value chain actors in these settings demonstrate a concern for safety by employing informal risk mitigation strategies. A “three-legged stool” approach was proposed, focusing on enabling environments, training, and behavioural incentives, as a cost-effective pathway to improve the FSC in such markets. However, significant differences persist between formal and informal markets, particularly in their ability to implement and standardise safety protocols [30].

A direct correlation exists between corporate social performance (CSP) and variables like national culture, geographic location, and economic development level [31]. The average CSP scores of European businesses are higher than those of North American companies, while Asian businesses, while still lagging behind Europe and North America, are ahead of developing nations. This variance is explained by the innate value systems that are moulded by national cultures and impact perceptions of corporate performance and responsibility. For example, an important factor is the regulatory environment, where European nations enforce more stringent regulations than the United States, which takes a more self-regulatory stance [31]. The national cultural factors that shape an organisation’s culture, which ultimately affects its FSC, are given below:

The idea of **individualism versus collectivism** is linked to the independent values of society. Individualistic cultures place more value on an individual’s standards and desires compared with collectivist cultures, which focus more on the needs of their social group. Owing to their alignment with protecting group interests, imposed standards may be more acceptable to workers in collectivist cultures. Furthermore, in contrast to individualistic cultures where personal knowledge and experience are more valued, collectivist cultures are more receptive to normative influences like team climate. It is imperative to comprehend these cultural aspects in order to apply and uphold food safety regulations in a variety of organisational contexts [27]. For instance, Asian cultures typically have more collective societal structures, so there is less critical examination of corporate practices. Western cultures, on the other hand, are more likely to value equality and corporate transparency because of their individualistic nature [31].

Food safety practices are significantly shaped by future orientation, which is the degree to which an organisation or a leader values long-term planning and delayed gratification. Long-term objectives and plans are more likely to be prioritised in cultures that place a high value on future orientation, which is frequently associated with greater societal well-being and economic prosperity [32].

Gender egalitarianism is a concept that promotes equal opportunities and roles for all people, regardless of gender. Its goal is to reduce gender-role differences. This has an impact on organisational performance. Higher degrees of gender equality are frequently found in organisations that have advanced human development [32].

The degree to which an employee of an organisation accepts ambiguity and uncertainty is known as uncertainty avoidance. High-uncertainty-avoidance cultures value environments with clear rules and structure. This characteristic influences how organisations approach risk management and food safety since it is generally linked to increased life satisfaction and quality of life [32].

Safety procedures are impacted by power distance, which reflects an organisation’s acceptance of hierarchy. Team members in high-power-distance cultures may be less receptive to feedback and more likely to follow instructions, even if they go against safety protocols. This can make it difficult for them to communicate and ensure safety compliance. Low power distance, on the other hand, is linked to greater human development, equitable resource distribution, and economic prosperity; these factors foster an atmosphere that is favourable for safety communication and protocol adherence [32]. In a diverse organisational setting, developing a positive safety culture requires an understanding of power distance.

A culture that values innovation, high standards, and improvement is said to be performance-oriented. Achievement and competition are given top priority in cultures that have a strong performance orientation. This orientation, which places a strong emphasis on results, effectiveness, and individual accountability in organisations settings, can have a strong impact on organisational performance [32].

The changing nature of the workforce in the food industry makes it more important than ever to adapt FSC models. An important part of Irish manufacturing, this sector has been facing a lack of skilled workers at different operational levels, which has led to the hiring of a culturally diverse workforce that includes contract workers and immigrants. Maintaining food safety standards is made more difficult by this diversity, as language barriers may cause important safety instructions to be misunderstood or misinterpreted [33] (pp. 13).

Globally, there is ample evidence linking improper food handling procedures to foodborne illnesses. Research has shown that inadequate cooking, reheating, and improper storage are the main causes of foodborne outbreaks. This emphasises the need to have the right information and skills about food safety, such as knowing the critical limits and being aware of allergens, as well as the significance of never working when ill. Safeguarding food production is imperative not only in Ireland but worldwide [34].

Even though these needs are acknowledged, food handlers’ levels of training vary widely. For example, a sizable proportion of respondents in the larger industry claim to have received no food safety training, despite the fact that a high percentage of food handlers in certain sectors reportedly have received substantial training. This disparity draws attention to a serious problem with the way the industry handles food safety training. In addition to ensuring legal compliance, food business operators are legally required to guarantee adequate training, which has an impact on the food businesses’ efficiency, reputation, and commercial success. The World Health Organization is in favour of basic education about food safety, with a focus on the “Five Keys to Safer Food” as the cornerstone of such courses [34].

However, the shift from knowledge to practice is still very difficult. Research shows that although understanding food safety is important, it does not always translate into appropriate food safety behaviour [35]. Training frequently concentrates on knowledge transfer, evaluation, and certification without sufficiently addressing the application of this knowledge in real-world scenarios, especially during times of high pressure. The fact that the workforce in the Irish food industry is made up of people from a variety of backgrounds makes this knowledge gap especially problematic.

In addition to knowledge-based training, behavioural training is required to address these issues. It has been demonstrated that behavioural training works better at maintaining positive behaviours over time. It focuses on raising the awareness of the significance of food safety, impacting normative views, attitudes, and the perception of behavioural control over food safety procedures. This method is especially useful in multicultural settings where training needs to be customised to fit various roles, languages spoken, and educational backgrounds in the food industry [35].

Thus, adopting FSC models necessitates a thorough approach. Language and cultural barriers should be addressed, the behavioural aspects of training should be given more attention, and training techniques should be tailored to the different learning styles and backgrounds of participants. Such a strategy is essential for the general prosperity and sustainability of the food processing industries in Ireland, as well as for guaranteeing food safety and public health. These adjustments are becoming more and more necessary as the industry becomes more globalised to uphold strict guidelines for food safety and avoid foodborne infections.

## 4. Organisational Commitment and Leadership

Leadership and organisational commitment are critical components of a culture of food safety. This is demonstrated by the high rates of noncompliance with food safety management regulations, which raise serious safety concerns and quality problems. There is a strong behavioural component to food safety, according to reports, and handler errors have been linked to numerous outbreaks in nonmanufacturing food businesses. This demonstrates how leadership and the overall work environment have a significant impact on food safety, which is deeply ingrained in the culture of an organisation. Establishing a culture where food handlers are equipped, encouraged, and mandated to maintain high standards of hygiene requires effective leadership [24].

### 4.1. Leadership Involvement in Food Safety

Effective leadership plays a crucial role in establishing a strong FSC, which in turn influences employee dedication, productivity, and compliance with safety procedures. Psychological attachment and employee retention are vital in today’s competitive environment. Good leadership increases employee commitment, which increases output and company performance [36].

FSC is strongly impacted by both transformational and transactional leadership approaches. With a focus on rewards and penalties, transactional leadership establishes clear performance objectives and guarantees adherence to food safety procedures. Research has provided evidence for this strategy, showing how transactional leadership in a food manufacturing company increased compliance by implementing formalised reward structures [37]. On the other hand, transformational leadership encourages a proactive FSC by inspiring staff members to exceed expectations and adopt food safety best practices. According to studies, transformational leadership in the restaurant sector stimulated staff involvement and continuous development, which reduced incidents involving safety [38].

A well-rounded strategy that incorporates both leadership approaches improves FSC. Transactional components, such as incentives and performance measures, guarantee adherence, whereas transformational leadership fosters a shared accountability and innovative culture. According to research, companies that adopt these styles boost employee satisfaction and compliance while achieving superior food safety outcomes [39].

Using both leadership philosophies, using behavioural strategies, and encouraging good communication are all necessary for effective management. Managers can foster a company culture where food safety is firmly ingrained, improving performance and lowering safety occurrences, by matching leadership with FSC goals [37].

### 4.2. Organisational Commitment

Effective organisational ownership and commitment are necessary for a strong FSC, as demonstrated in previous research. Employee motivation, work performance, and general involvement in following food safety procedures are all greatly impacted by this commitment. Building and maintaining a strong FSC within an organisation requires acknowledging the importance of employee commitment [27].

The impact of Employee behaviour on the culture surrounding food safety is a complex issue that involves both individual and organisational commitment. Research indicates that new employees often take up the dominant behaviour in the company, which can have a positive or negative impact on the culture surrounding food safety, depending on the dominant beliefs and behaviours [22] (pp. 31–41, 71–72). This emphasises the significance of identifying and supporting positive employee behaviours for businesses, particularly for those who mentor new hires.

**Figure 3:**
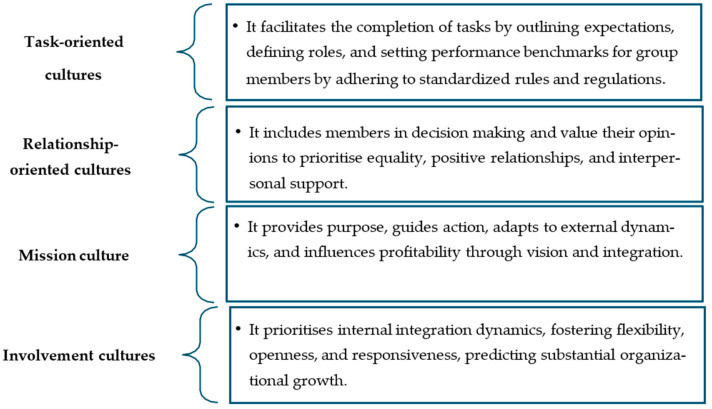
Types of organisational cultures demonstrated by leaders [39].

The behaviour of the employee is greatly affected by the different types of culture (Figure 3) in an organisation. The advantages of having both a mission culture and an involvement culture in an organisation have been noted [39]. Employees in these cultures not only support the objectives of the company but also improve their teamwork abilities. Similarly, various organisational culture profiles, whether relationship- or task-oriented have a strong impact on how employees behave. Relationship cultures place a strong emphasis on cooperation, idea generation, decision making, and effective communication, all of which have a positive impact on the culture of food safety [39].

Furthermore, in the context of food safety, employee behaviour is greatly influenced by the idea of employee commitment, particularly affective, normative, and continuance commitment. Normative commitment, which is duty-driven, and continuance commitment, which is motivated by the costs of leaving, are contrasted with affective commitment, which occurs when employees are driven and willing to exceed their duties. The latter might result in little effort being made merely to keep one’s job. Studies reveal that behavioural changes are more likely to be positively impacted by affective and normative commitments [39].

Putting in place effective communication strategies such as strong programs for rewards and recognition helps strengthen the positive behaviours related to food safety. These initiatives strengthen the bonds of trust between employees and upper management, which fosters a positive workplace environment. Consequently, there may be fewer incidents involving food safety and decreases in staff turnover. To keep these programs effective, regular assessment and adjustment are necessary [40].

To be effective, learning programs need to have objectives that are specific to roles and involve learners in the planning and implementation process. It is essential to modify learning plans according to the languages, literacy levels, cultural norms, and learning preferences of employees. To improve the retention and application of food safety knowledge, it is crucial to engage with concepts in real-world settings outside of the classroom [41].

These groups play a crucial role in promoting food safety. They take on the role of unofficial leaders and have a strong commitment to education and food safety. They play a crucial role in encouraging food safety procedures and bringing about change. A comprehensive approach to food safety requires diverse membership in these coalitions, representing different levels and functions within the organisation [41].

Posters, symbols, and slogans: It is critical to effectively communicate information about food safety using a variety of media, such as signs, posters, and symbols. These instruments ought to be easy to use, strategically positioned, and communicate desired behaviours in simple terms. Talking to staff members about food safety also facilitates a two-way flow of ideas and concerns [22] (p. 51). Setting measurable, quantifiable, risk-based, and precise goals for food safety is essential. This ought to be combined with suitable penalties in order to motivate behaviour. Combining leading and lagging indicators makes it easier to assess the overall performance of food safety [22] (pp. 57–65).

Improving FSC via resource management and teamwork is a complicated and multidimensional problem. According to a study, operational environment and human factors play a role in shaping FSC in addition to managerial and technological factors [42]. This implies that effective FSC requires a comprehensive strategy that takes into account both the operational environment and human factors [11].

**Figure 4:**
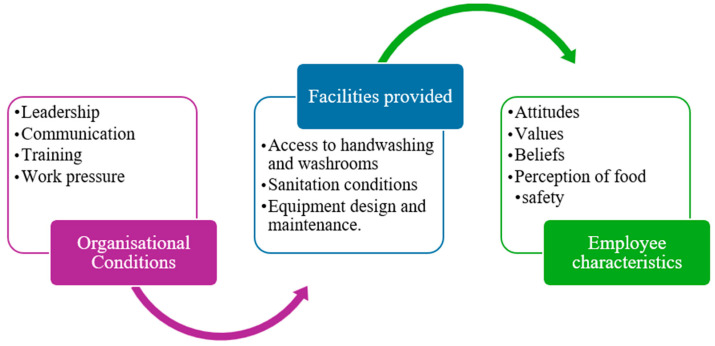
Factors influencing FSC [11].

According to Ungku Fatimah et al. (2014), an organisation’s FSC can be greatly impacted by operational features such as management systems, company size, product type, environment, and strategy [43]. To define the internal company environment, the study combined these company characteristics with those related to food production (Figure 4). Organisational conditions, organisational priority, and support facilities provided to the employees and the communication policy are some of the organisational factors that have been found to influence food safety practices among individual employees and at the organisation level. This approach highlights the importance of considering specific operational contexts when assessing FSC. However, poor resource management practices, such as the underfunding of protective gear, training, and incentives, can have a detrimental effect on food handlers’ opinions of the importance of food safety and hygiene.

Protective gear, commitment, and training are all indicators of food handler behaviour [11]. Furthermore, disparities in support functions like engineering and accounting can lead to the formation of subcultures within the organisation, potentially opposing, supporting, or interacting with the prevailing FSC. This suggests that promoting a positive FSC requires the careful consideration of departmental disparities and efficient resource management [11].

## 5. Challenges Faced by Organisations in Maintaining a Positive FSC

Sustaining a favourable FSC involves numerous obstacles that are interrelated and frequently exacerbate one another. One of the main challenges is the absence of a generally agreed definition for “safety culture”, which leads to misunderstandings between “safety climate”, which concentrates on the psychological attitudes of employees, and “safety culture”, which deals with situational and behavioural factors in businesses [44]. This ambiguity makes it more difficult to implement food safety protocols in an efficient manner, especially in organisations where safety norms may differ due to varied cultural backgrounds.

Food handler training programs are vital, but they frequently do not result in long-lasting behavioural change [35]. Despite receiving extensive training, staff members could nevertheless adopt ingrained habits that are representative of the company culture at large, which might not put food safety first. This problem highlights how difficult it is to apply theoretical understanding to real-world, risk-aware behaviour. In order to effectively implement a culture shift towards food safety and to reinforce the training, a supportive work environment is important [44].

According to studies, an organisation’s safety culture is greatly influenced by the dedication of senior management to food safety [12]. Prioritising profit over safety creates a toxic culture where safety is not prioritised. To complicate matters further, the diversity of ethnic backgrounds seen in food enterprises involves a variety of cultures and behaviours that might not conform to established food safety protocols [44].

These difficulties are made much more difficult by financial limitations, which especially affect small- and medium-sized enterprises (SMEs). Adherence to food safety regulations necessitates significant resources, such as consistent training and meticulous recordkeeping. Compliance may appear burdensome to SMEs due to the cost and complexity of regulatory obligations. Research highlights that operational constraints, such as staffing shortages and resource constraints, make it harder to maintain uniform food safety procedures [33] (pp. 2, 12–23).

Financial limitations exacerbate the difficulty of attaining and sustaining a good FSC. Overall, these issues are interconnected. In order to address these problems, a thorough strategy that unifies organisational safety culture and finances with efficient safety procedures is needed.

## 6. Comparative Analysis

The majority of the research so far has focused on the internal dynamics of FSC. Research has repeatedly shown that businesses handling high-risk products have a more proactive financial culture. Studies have shown that companies that manufacture high-risk products like meat and dairy follow strict guidelines for food safety [20]. This increased awareness is ingrained in their organisational culture and is more than just simple compliance. Empirical studies further support the relationship between product riskiness and the degree of FSC. According to these studies, organisations that are more vulnerable in terms of their supply chain, processes, and products need strong organisational support in order to guarantee that decisions about food safety are made consistently [45].

On the other hand, little is known about how outside influences, in particular the national context and food safety governance, shape FSC. The FSC within organisations is greatly influenced by the features of the regulatory environment and enforcement strategies. This theory is especially pertinent to transition economies since there is frequently a lack of developed food safety laws and enforcement procedures 11. Regulatory frameworks in transition economies often face challenges such as poorly defined laws, political influence, and limited enforcement capacity. These issues hinder the effective implementation of food safety management systems and, by extension, FSC [46]. To effectively develop strategies for food safety, it is imperative to comprehend the intricate interplay between internal and external factors. This comprehensive strategy can aid in improving food safety outcomes in a variety of settings [47].

Additionally, the significance of the comparative analysis of the FSC across various sectors can be understood based on studies [22] (pp. 57–65). Using a multimodal approach to assess FSC, De Boeck et al. (2016) covered farm butcheries and affiliated butcher shops in the meat industry [22] (pp. 57–65). This involved using a variety of samples, including meat, environmental, and hand swabs, to evaluate the perception of the climate surrounding food safety, the efficacy of the FSMS, and microbiological hygiene. One noteworthy finding was that centrally managed butcher shops scored higher on food safety climate evaluations than smaller, independent farm butcheries, especially when it came to leadership and communication. This result suggests that central management could promote improved hygiene standards and more effective FSMS implementation. The study also emphasised how crucial employee attitudes and actions are in forming FSC. Improved microbiological results were linked to a well-organised FSMS and a favourable food safety climate, emphasising the significance of internal culture in controlling food safety risks [42].

The FSC of an Irish dairy processing facility was evaluated using semi-structured interviews and questionnaires. The study revealed a generally stable or rising FSC in the industry. Although peer involvement was perceived as a potential obstacle, employee ownership was found to be highly effective. The study also revealed perception gaps between senior management and staff regarding the obstacles to FSC, with performance accountability for food safety standing out as a key area of concern [27]. These results showed that although the FSC was commendable overall, there was room for improvement in areas like peer involvement and clear communication. Thus, in order to promote a strong FSC, a dairy industry study emphasised the need for effective communication and involvement at all organisational levels [27].

Such comparative analyses are crucial because they offer a nuanced understanding of the distinct operational contexts and challenges that affect the FSC in each industry. For instance, the meat industry’s emphasis on centralised management highlights the value of structured systems and strong leadership in ensuring food safety [22] (pp. 57–65). Conversely, the dairy sector highlights the importance of thorough employee engagement and effective communication strategies [27]. These industry-specific variations imply that general strategies to improve FSC might not work. Such examples highlight that customised approaches tailored to the unique operational and cultural dynamics of each industry are essential for achieving effective and sustainable food safety practices.

By examining how various sectors approach FSC, we can identify best practices that could be adapted and applied across the industry. For instance, the seafood sector makes use of blockchain for traceability in the seafood, which offers lessons for enhancing transparency and accountability [48]. This can be of importance in the fresh produce sector for ensuring the freshness of products and traceability [49] (pp. 86–87). Similarly, the use of precision monitoring technologies in seafood food processing such as IoT sensors to monitor temperature in real time during storage and transport is a common practice in the seafood sector, which could inform better control and safety measures in fresh produce handling and processing.

Additionally, comparing training initiatives across sectors reveals opportunities to innovate in employee engagement and food safety knowledge. Effective training considers company culture, sets clear goals, and measures outcomes to enhance FSC [50]. Continuous improvement in training design and implementation is crucial. Insights from various sectors can lead to more effective, engaging, and inclusive training approaches that are applicable industry-wide.

Furthermore, by using a comparative approach, it becomes easier to comprehend how different employee behaviours, management views, and operational procedures vary among industries and affect food safety. These kinds of insights are essential for creating more potent interventions to improve FSC, which will ultimately raise the calibre and safety of the food produced in these sectors.

## 7. Conclusions

FSC has become a cornerstone of modern food safety management, evolving from a focus on compliance to a deeper integration as an organisational value. The effectiveness of FSC relies heavily on strong leadership, particularly transformational leadership, and the commitment of the organisation. Cultural diversity within the workforce presents unique challenges, requiring tailored strategies that align with both national and organisational cultures. Sector-specific analysis reveals that high-risk industries tend to maintain a more robust FSC due to stricter regulatory demands, while others may struggle with communication and employee engagement. To strengthen FSC, it is crucial to incorporate behavioural training that considers these cultural dynamics. Ultimately, a comprehensive and context-sensitive approach involves developing strategies that holistically address industry-specific challenges, cultural nuances, and organisational goals. This approach combines behavioural insights, effective leadership, and tailored training programs to ensure that food safety practices resonate across diverse regulatory and operational landscapes, ensuring the ongoing sustainability and success of food processing industries in diverse regulatory landscapes.

## Data Availability

No new data were created or analysed in this study. Data sharing is not applicable to this article, as it is a review of the existing literature.
